# Clinical Evaluation of a 1060‐Nm Laser for Non‐Invasive Fat Reduction of the Abdomen and Coulotte/Thighs in Men and Women

**DOI:** 10.1111/jocd.70558

**Published:** 2025-11-29

**Authors:** Paolo Bonan, Irene Fusco, Francesca Madeddu, Tiziano Zingoni, Shady Mahmoud Attia Ibrahim

**Affiliations:** ^1^ Laser Cutaneous Cosmetic & Plastic Surgery Unit Villa Donatello Clinic Florence Italy; ^2^ El.En. Group Calenzano Italy; ^3^ Department of Dermatology, Faculty of Medicine Al‐Azhar University Cairo Egypt

## Abstract

**Background:**

There is a growing need for a more visually pleasing silhouette through non‐invasive body contouring procedures.

**Aim:**

This study evaluates the use of a 1060‐nm laser system equipped with a temperature detector, for non‐invasive fat reduction of the female and male abdomen and female culottes and thighs, which represent the prevalent areas among individuals requiring cosmetic contouring.

**Material and Method:**

A total of 20 male and 40 female patients who were considering lipolysis without invasive procedures of the abdomen and coulottes/thighs areas were treated with a 1060‐nm laser system. All patients underwent 4 laser treatment sessions spaced at least 4–6 weeks apart. The study's endpoints were photographic investigations by independent blinded physicians at 3 months follow‐up, screening for adverse effects, abdomen and coulotte/thighs circumference measurements, BMI, and 4‐point Global Aesthetic Improvement Scale (GAIS). Clinical measurements were evaluated before (T0) and at 3 months follow‐up after the last laser treatment session (Tf).

**Results:**

During the entire laser treatment, the skin temperature remained below 40°C, demonstrating the safety of the study device in preserving the skin from thermal damage. Clinician assessment from the photographic investigation, GAIS, BMI and circumference mean values showed good efficacy of laser treatment and visible aesthetic results for abdomen and coulotte/thighs areas both in men and women.

**Conclusions:**

The use of 1060 nm lasers offers an alternative therapy choice for patients who demand a non‐surgical, non‐invasive procedure for fat reduction with no serious consequences.

## 1 Introduction

The need for a more visually pleasing silhouette has existed throughout history [1], and as technology has advanced, along with it, the need for body sculpting operations has changed. Liposuction has long been the standard treatment for individuals with localized areas of undesirable fat, such as the flanks, thighs, or abdomen, and it remains the most popular body sculpting procedures [2, 3].

Although surgery can result in remarkable clinical improvements, there is significant accompanying postoperative rehabilitation and financial cost [4].

Liposuction was the most used method for fat reduction and body shaping; nevertheless, associated risks such as post‐operative problems, recovery, and downtime can occur [5, 6].

Many patients consider non‐invasive or minimally invasive procedures with quick recovery and minimal risk of adverse reactions to be preferable if the clinical result is evident. Alternative methods for localized fat elimination [7] include lasers [8], high intensity–focused ultrasound (HIFU) [9, 10, 11] radiofrequency (RF) devices [12], and selective cryolysis [13, 14].

With these technologies, the thickness of the fat layer gradually decreases over the course of 3 months after treatment; this is especially noticeable in individuals who have small, distinct fat bulges [15].

While laser lipolysis uses laser energy to disintegrate fat cells with the use of cannulas and fibers to be inserted into the adipose tissue, cryolipolysis employs extremely cold temperatures to freeze fat cells. Procedures for cryolipolysis fat reduction usually take longer than laser lipolysis. Although both methods are effective at reducing body fat, laser liposuction, which also tightens the skin, is thought to be more suitable for areas that need both fat reduction and skin enhancement [16].

Thermal energy is delivered to the skin's deeper layers by both radiofrequency and focused ultrasonic lifts. As opposed to radiofrequency, which works better on wider areas, ultrasound works best on particular areas like wrinkles. Laser liposuction with skin‐tightening effects may be more beneficial for areas that require both fat removal and skin improvement.

Laser assisted liposuction, increases the frequency of use by selectively heating fat areas with fewer incisions. In fact, compared to other liposuction techniques, laser lipolysis has a number of benefits, including reduced bleeding, less discomfort and swelling, skin tightness, no incision and repair required, less tissue damage, and an earlier recovery time [17, 18, 19]. Specifically, lasers cause damage to the fat area as the energy is absorbed by the aqueous chromophores and fat cells, where it is then transformed into heat [20, 21].

Specifically, the wavelength of 1060 nm was proven to be effective in transmitting laser energy to the subcutaneous target through the skin. Furthermore, due to its low affinity for melanin, scientific investigations have shown that this wavelength is safe to use for treating dark skin [22]. When compared to other visible to infrared wavelengths, fat's high penetration depth generates heat over a greater volume without producing hot spots. Published preclinical and clinical research has already shown that the hyperthermic 1060‐nm laser therapy is safe and effective for non‐invasive fat removal [22, 23, 24, 25, 26, 27].

The 1060 nm wavelength with pulse duration longer than the thermal relaxation time of the dermal and epidermal layers and annex has no specific chromophore in the skin and therefore generates non‐specific heating and penetrates the subcutis with very limited downtime [28].

Adipose tissue that is at least 2 cm thick is heated to the therapeutically relevant target temperature at depths of 1 to 3 cm, according to in vivo temperature measurements using a thermocouple needle. The depth of the thermal effect in the tissue of this treatment design is significantly greater than the depth of optical penetration alone because of thermal conduction. Consequently, the thermal effect of laser radiation is related to heat conduction [29].

The 1060 nm laser's main mechanism of action for lipolysis is heat, which accelerates the local disintegration of fat cells. Triglycerides are broken down by this rise in temperature into glycerol and free fatty acids, which are then transported by fatty acid carriers out of the cell. Following this event, they enter the blood circulation and link to albumin, allowing them to circulate throughout the body to be eliminated by cells as needed [25].

In this context, non‐invasive laser systems have been developed to provide energy from external sources using applicators that are placed on the skin for a few minutes [30, 31].

In the preliminary report of Rothaus and colleagues following the treatment with the subject device all patients showed a significant decrease in subcutaneous tissue thickness (SQR) while increasing voluntary muscle thickness (VMI). In addition, the clinical results of the study of Mezzana et al. [31] show that laser treatment with a heating temperature of 42°C–47°C that destroys adipocytes resulted in a considerable reduction in flanks, abdomen, internal legs, trochanter buttocks and arm circumference, leading to a harmonic reconstruction of patients' body shapes. However, the development on the market of non‐invasive laser device procedures which preserve the epidermis during therapy is an important concern. As a result, it is critical to design laser systems fitted with a temperature detector capable of monitoring the skin's temperature during the whole treatment period.

In view of this consideration and already published scientific data, the current study assesses the use of a 1060‐nm laser system equipped with a temperature detector, for non‐invasive fat reduction of the female and male abdomen and female coulotte and thighs, which represent the prevalent areas among individuals requiring cosmetic contouring.

## 2 Materials and Methods

### 2.1 Patient

A total of 20 male and 40 female patients who were looking for non‐invasive lipolysis of the abdomen and coulottes/thighs areas, who expected to abstain from adopting significant modifications in their dietary habits or lifestyle throughout the test period, with a body mass index (BMI) of 30 or below, pinchable fat in the treatment area, and realistic expectations, were treated. Patients have been examined for excluding criteria, in accordance to the device safety guide and indications for use, including the presence of open lesions and wounds (treatment should only be administered to intact, healthy skin that shows no signs of impeded wound healing), the presence of an unrepaired abdominal hernia, pregnancy and/or breastfeeding, the presence of a tattoo in the treatment area, a history of skin photosensitivity disorders or taking photo‐sensitized medication, neuropathic disorder, diabetic neuropathy or impaired skin sensation, infectious diseases (especially hepatitis B and C), renal and hepatic insufficiency/dysfunction, subjects with diabetes (type I to decompensated type II), hypertriglyceridemia and hypercholesterolemia. Demographics details of recruited patients are reported in Table 1.

**TABLE 1:** Patient's demographic details.

Area	Abdomen	Coulotte/thighs	Abdomen
Number of patients	20	20	20
Sex	Women	Women	Men
Age	44.9 ± 7.3	37.7 ± 6.7	43.3 ± 7.6
Height (cm)	162.9 ± 9.3	161.7 ± 5.5	179.1 ± 6.1
BMI before (T0)	25.4 ± 1.9	23.0 ± 2.1	25.7 ± 1.1

All subjects provided informed consent.

### 2.2 Study Device and Treatment Protocol

All patients were treated with the study device (PHYSIQ 360, DEKA M.E.L.A srl, Calenzano, Italy) following a standard procedure. The device is equipped with four independent 1060‐nm laser applicators.

Each laser applicator has a cooled window that allows irradiating an area of 60 × 40 mm. This device is equipped with a skin temperature detector to further increase patient safety. If the area to be treated was large, it was possible to use multiple applicators that are held in position thanks to an elastic bandage that allows for hands‐free treatment.

Anesthesia is not required and was not utilized in this study as the device parameters must be set based on the patient's feedback and the perception of more or less intense heat is an additional tool to guarantee the patient's safety and prevent any unwanted effects. The laser energy is spread evenly throughout the surface of the applicator's treatment window, ensuring uniform energy density. This energy dissemination matrix was designed to provide a uniform supply of energy, enhancing comfort for patients and treatment security. All patients underwent 4 laser treatment sessions spaced at least 4–6 weeks apart. A transparent gel was applied before the treatment.

Based on an evaluation of each patient's unique treatment needs, the treatment area's location and dimensions were chosen, and each laser session was conducted with the same method. A total of 20 female abdominal areas, 20 male abdominal areas and 20 female Coulotte/thighs were treated.

Pre‐treatment care for patients includes avoiding direct sun exposure in the treatment area 7 days prior to treatment, keeping the skin free from makeup, lotions, creams and body oils, removing all jewelry and piercings pertaining to the treatment area, and always keeping the skin clean with minimal to no hair.

For abdominal treatment procedure patients were situated on their backs, leaving their abdomen exposed. and with the head of the bed raised at an angle of approximately 45°. Pillows were placed under the knees to assure the patient's comfort. Four applicators were uniformly placed along the abdominal circumference.

For Coulotte/thighs treatment procedure only 2 laser applicators were utilized and placed on the Coulotte/thighs sides. The patients were placed on the treatment bed to ensure that the belt is snug enough to prevent the applicators and frames from lifting off the skin.

The treatment time changes depending on the area to be treated and can last up to a maximum of 30 min.

The device used allows modifying the intensity of the Power delivered through 33 levels (with a maximum Power density of 1.4 W/cm^2^) which are chosen based on the feedback from the patient who must feel the following sensations: prickling, pinching, pressure, longer peaks of moderate deep heat and cooling.

For post treatment care the treated areas should be massaged twice daily for 5–10 min for the first 2 weeks. Patients should stay well hydrated and engage in light physical activity to help mobilize the destroyed fat for processing through the lymphatic system.

### 2.3 Clinical Measurements

The study's endpoints were photographic investigations by independent blinded physicians at 3 months (±1 week) follow‐up, screening of adverse effects, abdomen and coulotte/thighs circumference measurements, BMI, and 4‐point Global Aesthetic Improvement Scale (GAIS) (None: 0; Slight: 1; Mild: 2; Excellent: 3). Clinical measurements were evaluated before (T0) and at 3 months follow‐up after the last laser treatment session (Tf). The abdomen and coulotte/thighs circumference measurements were taken at the same distance from established reference points.

Patients were questioned about whether they had noticed any changes in the treatment region, when they had noticed them, how much they had changed, whether they had experienced any sensations during treatment, and how long the sensitivity persisted after treatment.

### 2.4 Statistical Analyses

The Student's paired *t* test was used to examine changes in clinical parameters between baseline and 3 months following the last laser treatment session. Quartiles of patients' abdomen and coulotte/thighs circumference were calculated separately for men and women. The mean ± standard deviation (SD) and confidence intervals are shown.

### 2.5 Side Effects

All possible side effects such as redness, pain, nodules, swelling/edema, hardness, bruising, itching, numbness, blistering/skin burn, skin contour irregularities, necrosis, changes in skin laxity were monitored during the entire treatment period.

## 3 Results

### 3.1 Clinical Measurements

**FIGURE 1:**
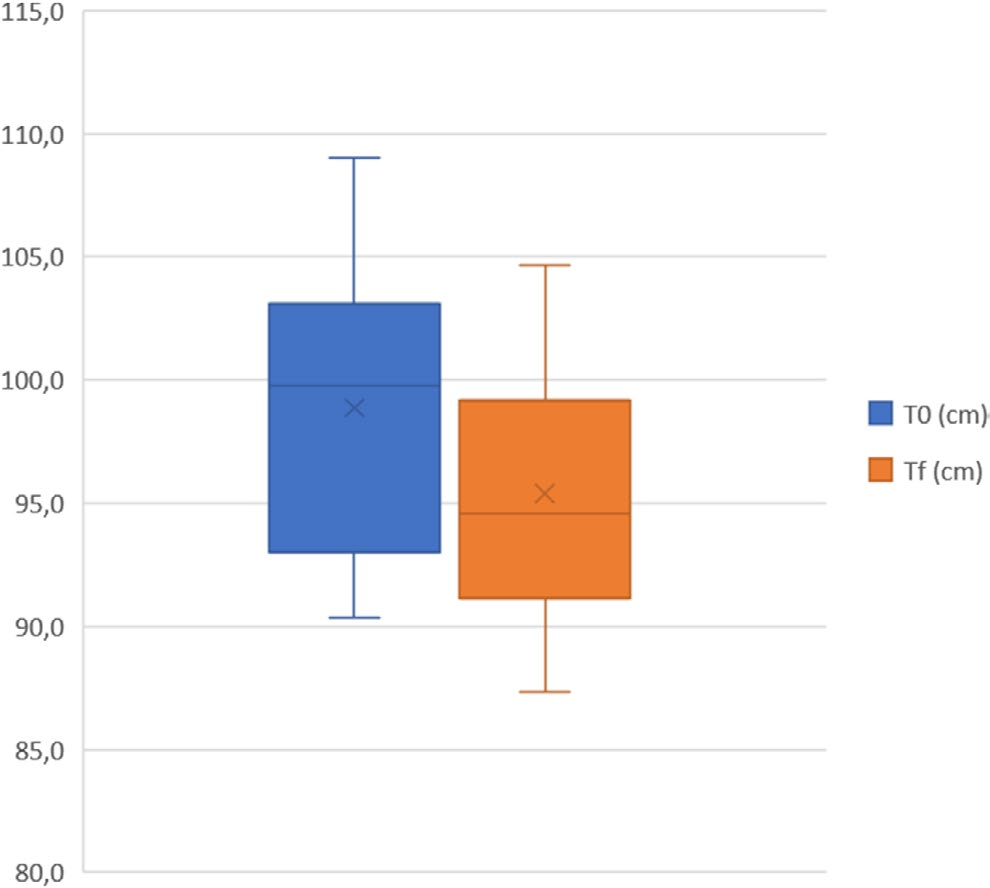
Quartile distribution of abdominal circumference (cm) measured before (T0) and 3 months after the last treatment (Tf).

**FIGURE 2:**
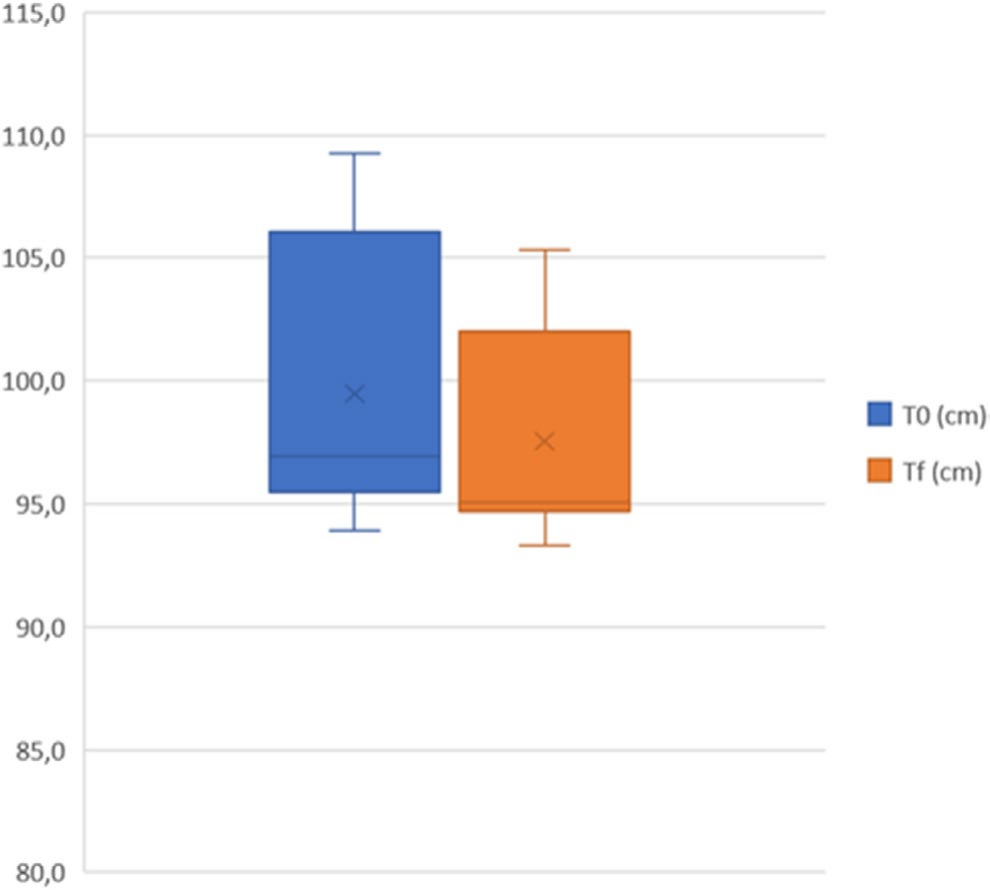
Quartile distribution of coulotte/thighs circumference (cm) in female subjects measured before (T0) and 3 months after the last treatment (Tf).

**FIGURE 3:**
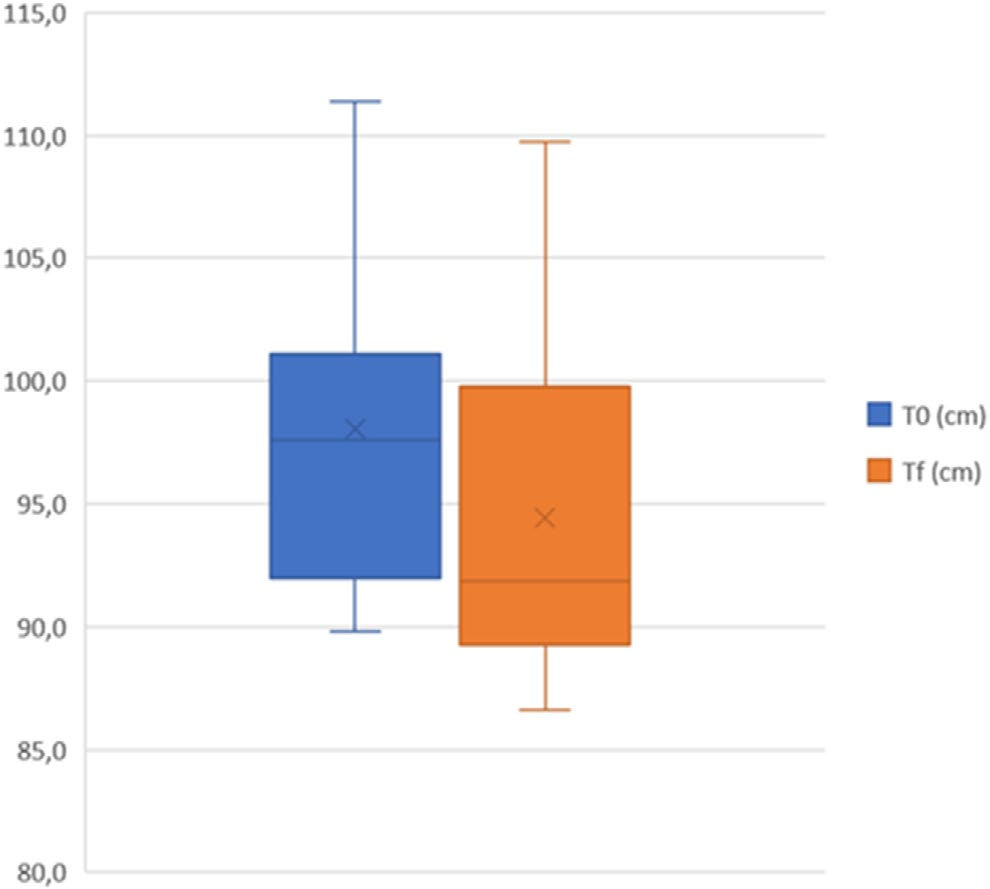
Quartile distribution of abdominal circumference (cm) in male subjects measured before (T0) and 3 months after the last treatment (Tf).

A total of 20 women with a mean age of 44.9 (±7.3) were treated on abdominal area, 20 women with a mean age of 37.7 (±6.7) were treated on coulotte/thighs area and 20 men with a mean age of 43.3 (±7.6) were treated on abdominal area. During the entire laser treatment, the skin temperature remained below 40°C, demonstrating the safety of the study device in preserving the skin from thermal damage. As reported in Table 2, female abdominal circumference mean value significantly (*p* < 0.001) decreased from 98.9 cm [SD:5.9; IC99.9%: 93.7−104.0] at T0 to 95.4 cm [SD:5.4; IC99.9%: 90.7−100.1] at Tf. Female coulotte/thighs circumference mean value significantly (*p <* 0.001) decreased from 99.5 cm [SD: 5.7; IC99.9%: 94.5 −104.4] at T0 to 97.5 cm [SD:4.2; IC99.9%: 93.9−101.2] at Tf. Male abdominal circumference mean value significantly (*p* < 0.001) decreased from 98.0 cm [SD: 6.3; IC99.9%: 92.5−103.5] at T0 to 94.4 cm [SD:6.9; IC99.9%: 88.4−100.5] at Tf. These results are graphically represented in Figures 1, 2, 3 as quartile distribution of mean values.

**TABLE 2:** Clinical measurements evaluated before (T0) and at 3 months follow‐up after the last laser treatment session (Tf).

Area	Abdomen	Coulotte/thighs	Abdomen
Number of patients	20	20	20
Sex	Women	Women	Men
Circumference (cm) before (T0)	98.9 ± 5.9	99.5 ± 5.7	98.0 ± 6.3
Circumference (cm) after (Tf)	95.4 ± 5.4 (*p* < 0.001)	97.5 ± 4.2 (*p* < 0.001)	94.4 ± 6.9 (*p* < 0.001)
BMI before (T0)	25.4 ± 1.9	23.0 ± 2.1	25.7 ± 1.1
BMI after (Tf)	25.3 ± 1.8	22.8 ± 2.3	25.6 ± 1.1
GAIS			
None	18%	11%	11%
Slight improvement	29%	39%	17%
Mild improvement	35%	39%	50%
Excellent improvement	35%	22%	33%

The mean value of BMI of women treated on abdominal area varied from 25.4 ± 1.9 at T0 to 25.3 ± 1.8 at Tf. The mean value of BMI of women treated on coulotte/thighs areas varied from 23.0 ± 2.1 at T0 to 22.8 ± 2.3 at Tf. The mean value of BMI of men treated on abdominal area varied from 25.7 ± 1.1 at T0 to 25.6 ± 1.1 at Tf.

**FIGURE 4:**
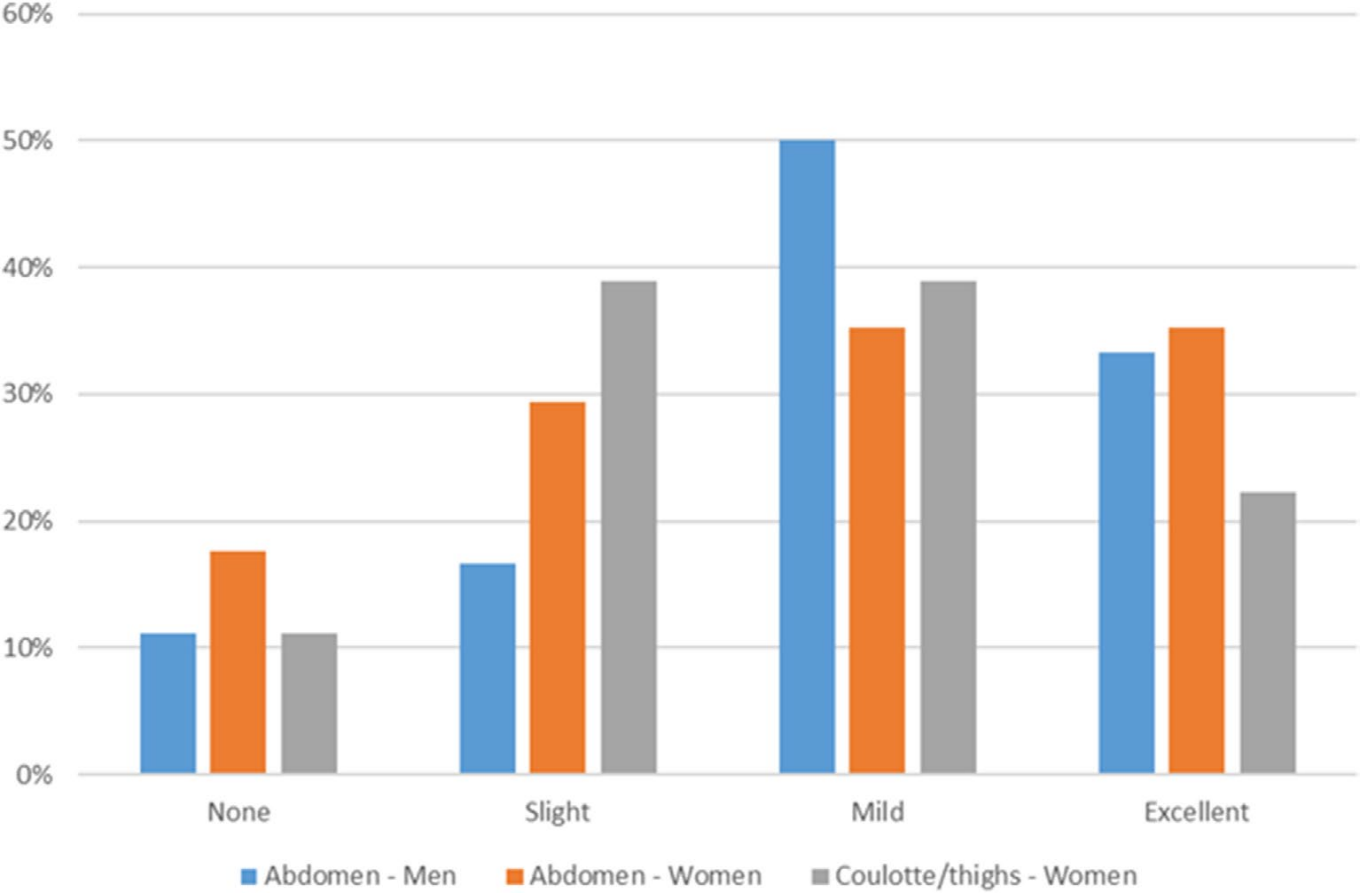
Results of the 4‐point Global Aesthetic Improvement Scale (GAIS) at 3 months after the last laser treatment session.

According to the GAIS scoring criteria 3 months after the last treatment session in the group of women treated on the abdominal area 18% of patients reported no improvement, 29% of patients reported slight improvement, 35% of patients reported mild improvement and 35% of patients reported excellent improvement. Among the women who were treated on the coulotte/thighs areas, 11% of patients reported no improvement, 39% of patients reported slight improvement, 39% of patients reported mild improvement and 22% of patients reported excellent improvement. Finally, in the group of men treated on the abdominal area, 11% of patients reported no improvement, 17% of patients reported slight improvement, 50% of patients reported mild improvement and 33% of patients reported excellent improvement (see Figure 4).

**FIGURE 5:**
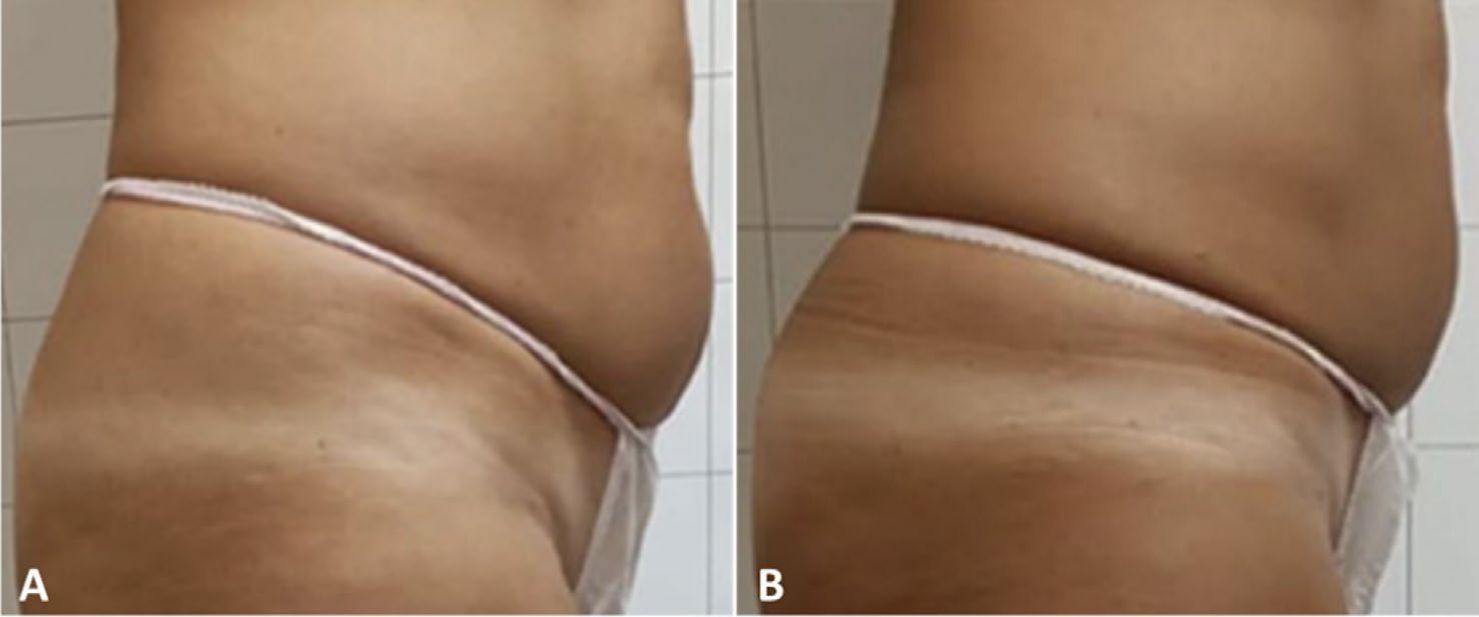
Lateral view of a female patient at baseline (A) and 3 months after the last abdominal treatment (B).

**FIGURE 6:**
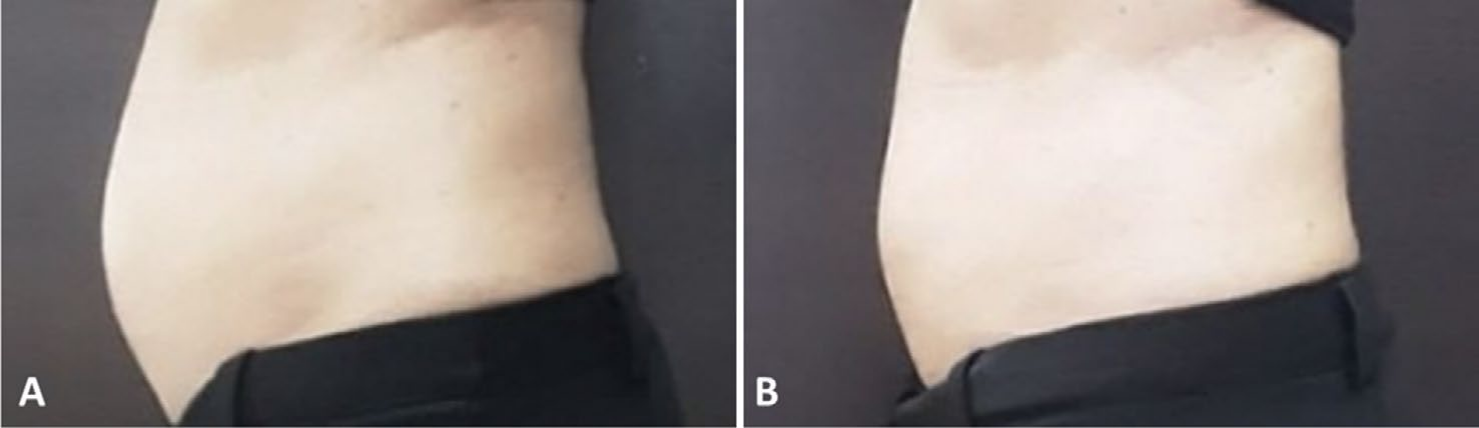
Lateral view of a female patient at baseline (A) and 3 months after the last abdominal treatment (B).

**FIGURE 7:**
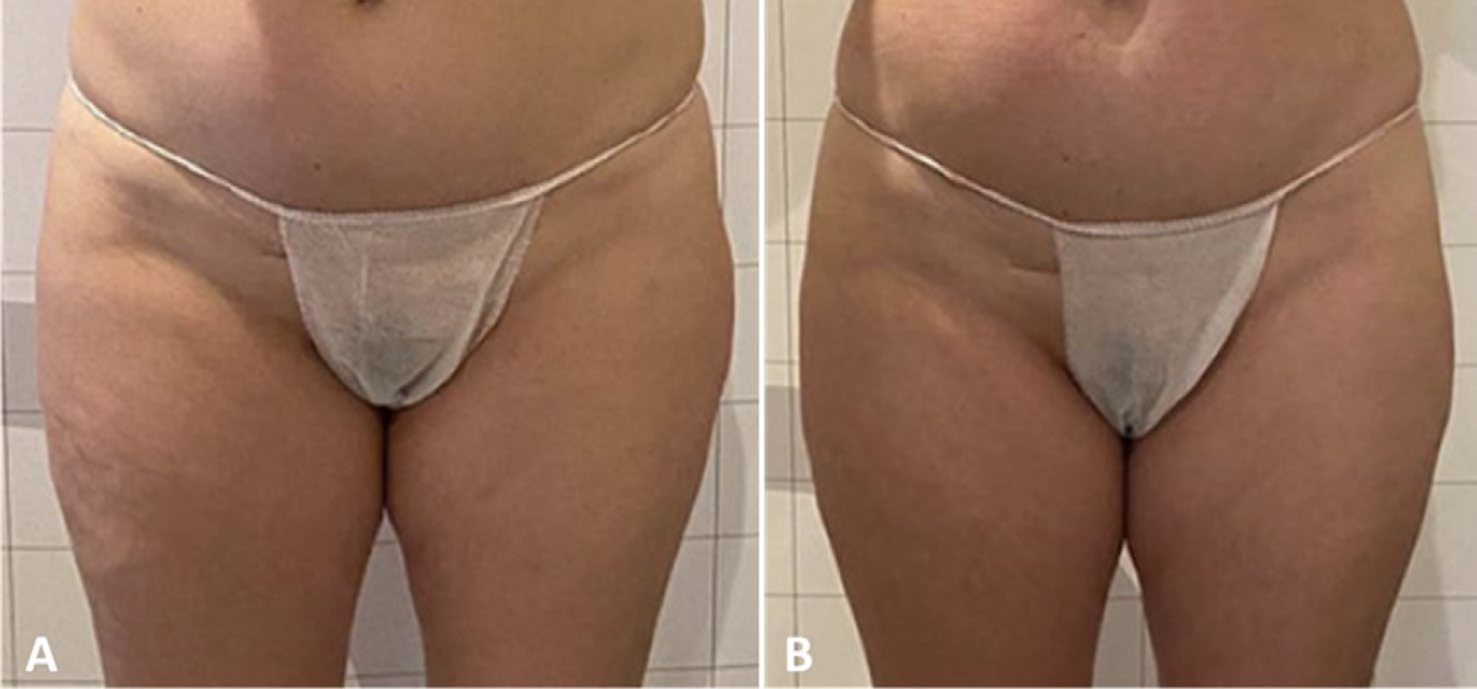
Front view of a female patient before (A) and 3 months after the last treatment on coulotte/thighs (B).

**FIGURE 8:**
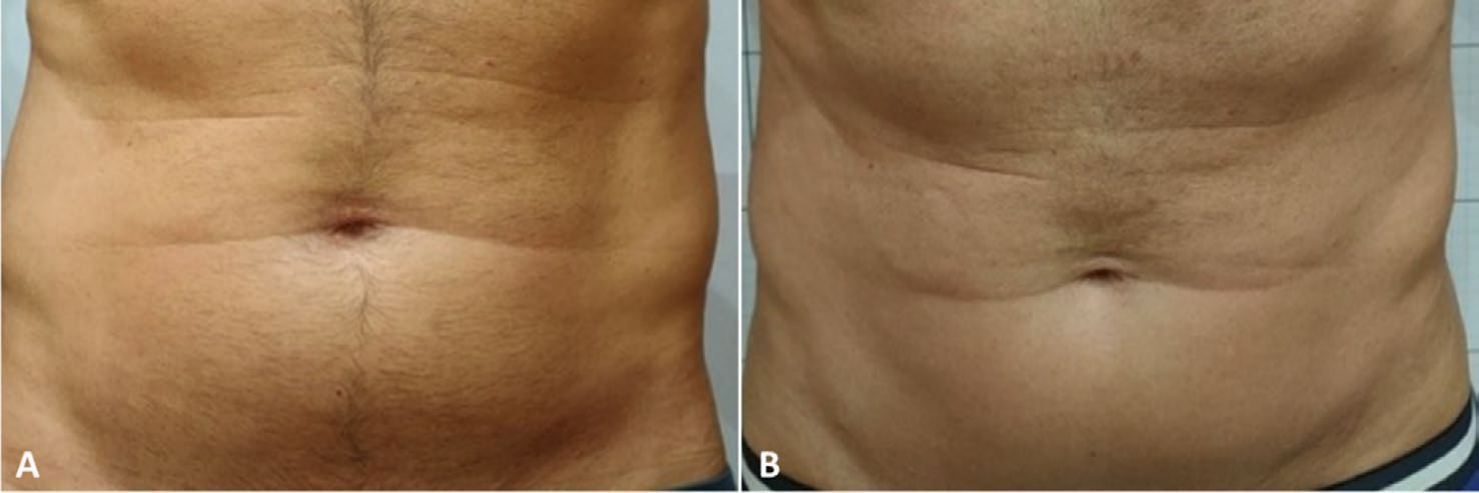
Front view of a male patient before (A) and 3 months after the last treatment on the abdomen (B).

Clinician assessment from the photographic investigation showed good efficacy and visible aesthetic results for abdomen and coulotte/thighs areas both in men and women. Figures 5, 6, 7, 8 show some clinical cases with favorable aesthetic outcomes.

### 3.2 Side Effects

Slightly pink or red skin immediately after treatment was observed and this phenomenon can last for hours. In 7 treatments (3%) a transitory erythema occurred which lasted 4 days and the application of cold gel compresses was suggested. In 4 treatments (1%), swelling and tenderness were observed which lasted about 2–3 weeks. In 16 treatments (6%) a firmness of the tissues or nodules was also observed. The nodules last 15 days and usually resolve on their own. In this case the patient was advised to have a massage until the next session. Finally, 2 cases (1%) of first‐degree burns, which resolved without hypo‐ or hyperpigmentation were observed.

## 4 Discussion

It is well known that adipocyte cells are damaged by hyperthermia, and a number of studies indicate that using a 1060 nm laser in conjunction with a surface cooling system may cause and sustain hyperthermia in the subcutaneous adipose tissue [32]. An inflammatory reaction and adipocyte injury could result after a 42°–47°C rise in tissue temperature. Adipocyte cells' viability has been demonstrated to drop by more than 80% when exposed to 50°C for brief periods of time. Additionally, in order to disrupt the integrity of the target adipocytes' cell membranes, which ultimately undergo cell death, a regulated temperature of 42°–47°C must be maintained [27]. Over the course of six to twelve weeks, at the conclusion of the apoptotic process, the body's immune response eliminates the cellular debris [22]. This research has revealed that the 1060‐nm laser applied externally to the skin of patients, represents a fast, safe, and efficient technique for eliminating excess body adipose tissue providing significant results.

Both female and male abdominal circumference mean values measured before and after laser treatment demonstrated a significant reduction of adipose tissue in this body area, which was confirmed by the photographic assessment. Additionally, following laser treatment, there was also a noticeable and significant decrease in the mean values of the female coulotte/thigh circumference.

These encouraging findings are further supported by GAIS analysis, which shows that most patients experienced mild to excellent improvements.

In line with our findings, the study of Kang and colleagues [33] reported that after 3 months, 74% of patients reported a clear reduction in fat in the flank area after using the 1060 nm laser.

A decreased fat layer results from the two to three month‐long process of apoptotic cell clearance [34].

According to the study's findings, this 1060‐nm laser system can be used to reduce subcutaneous fat in both the abdomen and coulotte/thighs areas. With comparatively little effort, laser technology may be readily modified to fit various sized and shaped treatment heads. Two benefits of this 1060‐nm laser system are its relatively short treatment time and hands‐free flexible applicator device, which allows treatment of up to four anatomical areas simultaneously. The small quantity of fat that is eliminated after each session is the only drawback of this device. Patients should have reasonable expectations regarding the restricted quantity of fat that can be eliminated, as this is true for the majority of non‐invasive fat reduction procedures. The safe transmission of laser radiation into the subcutaneous tissues through the skin is essential to this device's effectiveness. Consequently, the risk of burns is decreased by the cooling system that gets in contact with the skin. Indeed, only 2 cases (1%) of first‐degree burns, which resolved without hypo‐ or hyperpigmentation, were observed in this investigation, probably as a result of residual hair in the treatment area.

Finally, the presence of a temperature detector, which was absent in previous laser system [30, 31] permits the operator to monitor the patient's skin temperature in order to preserve the epidermis from thermal damage; the study system is equipped with a security mechanism that prevents the laser from activating if contact with the skin is interrupted or if the skin temperature is too high.

### 4.1 Study Limitations

The lack of a large sample size, patients' pain assessment and ultrasound investigation to evaluate fat thickness throughout the investigation. Indeed, without having patients under anesthesia or undergoing an intrusive procedure, ultrasonography measures are usually regarded as the gold standard for determining adipose thickness [35].

## 5 Conclusion

The use of 1060 nm lasers offers an alternative therapy option for patients desiring a non‐surgical, non‐invasive procedure for fat reduction with no serious consequences.

## Author Contributions

Conceptualization, P.B., T.Z., and S.M.A.I.; methodology, P.B., T.Z., and S.M.A.I.; software, P.B., F.M., T.Z., and S.M.A.I.; validation, P.B., T.Z., and S.M.A.I.; formal analysis, P.B., F.M., T.Z., and S.M.A.I.; investigation, P.B., I.F., F.M., T.Z., and S.M.A.I.; resources, P.B., I.F., F.M., T.Z., and S.M.A.I.; data curation, P.B., I.F., F.M., T.Z., and S.M.A.I.; writing – original draft preparation, I.F. and F.M.; writing – review and editing, P.B., I.F., F.M., T.Z., and S.M.A.I.; visualization, P.B., I.F., F.M., T.Z., and S.M.A.I.; supervision, P.B., T.Z., and S.M.A.I.; project administration, P.B. and T.Z.; funding acquisition, P.B. and T.Z. All authors have read and agreed to the published version of the manuscript.

## Ethics Statement

The article is in accordance with the principles of the Declaration of Helsinki on Ethical Principles for Medical Research involving human subjects. No activity was carried out outside the scope of the device's intended purpose or that no additional invasive or burdensome procedures were carried out compared to the procedure performed under the normal condition of use of the device.

## Consent

A written informed consent was obtained by all subjects involved in this study.

## Conflicts of Interest

Authors T.Z., F.M., and I.F. were employed by El.En. Group. The remaining authors declare that the research was conducted in the absence of any commercial or financial relationships that could be construed as a potential conflicts of interest.

## Data Availability

Data available on request from the authors.
